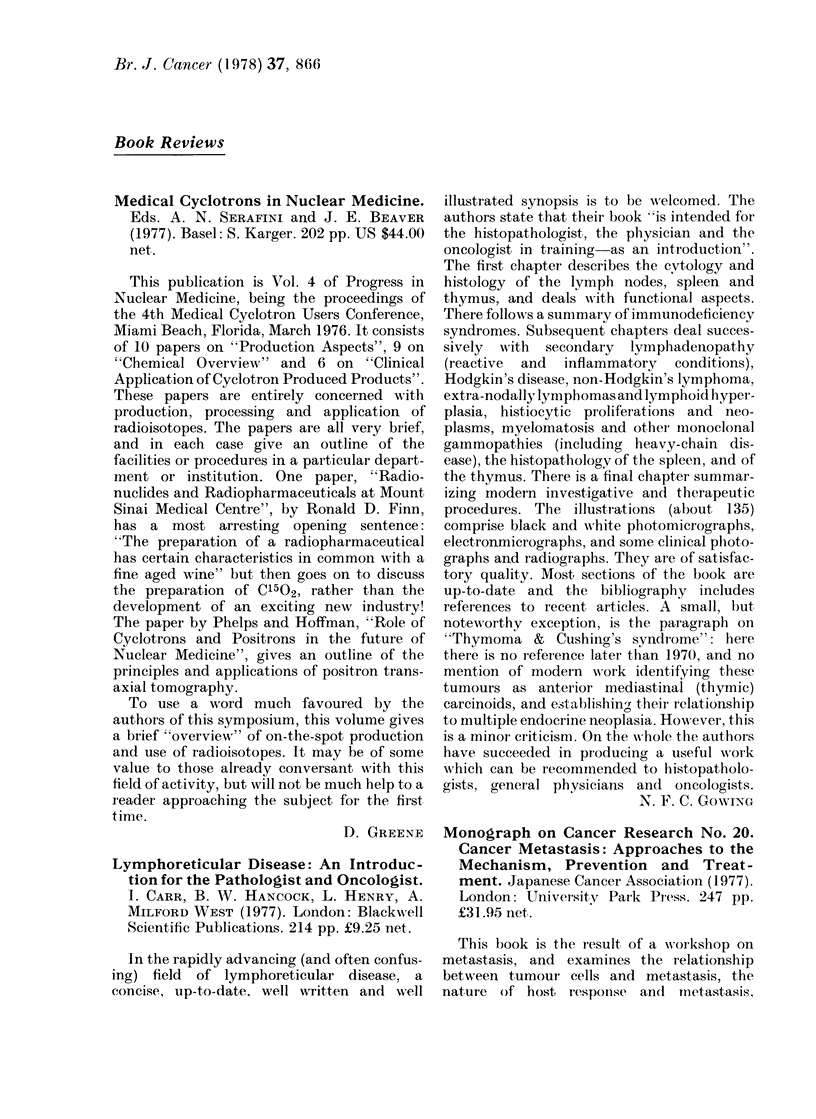# Medical Cyclotrons in Nuclear Medicine

**Published:** 1978-05

**Authors:** D. Greene


### *Book Reviews*

**Medical Cyclotrons in Nuclear Medicine.** Eds. A. N. SERAFINI and J. E. BEAVER (1977). Basel: S. Karger. 202 pp. US $44.00 net.

This publication is Vol. 4 of Progress in Nuclear Medicine, being the proceedings of the 4th Medical Cyclotron Users Conference, Miami Beach, Florida, March 1976. It consists of 10 papers on "Production Aspects", 9 on "Chemical Overview" and 6 on "Clinical Application of Cyclotron Produced Products". These papers are entirely concerned with production, processing and application of radioisotopes. The papers are all very brief, and in each case give an outline of the facilities or procedures in a particular department or institution. One paper, "Radionuclides and Radiopharmaceuticals at Mount Sinai Medical Centre", by Ronald D. Finn, has a most arresting opening sentence: "The preparation of a radiopharmaceutical has certain characteristics in common with a fine aged wine" but then goes on to discuss the preparation of $C^{15}O_2$, rather than the development of an exciting new industry! The paper by Phelps and Hoffman, "Role of Cyclotrons and Positrons in the future of Nuclear Medicine", gives an outline of the principles and applications of positron transaxial tomography.

To use a word much favoured by the authors of this symposium, this volume gives a brief "overview" of on-the-spot production and use of radioisotopes. It may be of some value to those already conversant with this field of activity, but will not be much help to a reader approaching the subject for the first time.

D. GREENE